# Extended Amygdala Neuropeptide Circuitry of Emotional Arousal: Waking Up on the Wrong Side of the Bed Nuclei of Stria Terminalis

**DOI:** 10.3389/fnbeh.2021.613025

**Published:** 2021-02-09

**Authors:** William J. Giardino, Matthew B. Pomrenze

**Affiliations:** Department of Psychiatry and Behavioral Sciences, Stanford University School of Medicine, Stanford, CA, United States

**Keywords:** bed nuclei of the stria terminalis, extended amygdala, neuropeptide, arousal, circuit, sleep, wakefulness, bed nucleus of stria terminalis (BNST)

## Abstract

Sleep is fundamental to life, and poor sleep quality is linked to the suboptimal function of the neural circuits that process and respond to emotional stimuli. Wakefulness (“arousal”) is chiefly regulated by circadian and homeostatic forces, but affective mood states also strongly impact the balance between sleep and wake. Considering the bidirectional relationships between sleep/wake changes and emotional dynamics, we use the term “emotional arousal” as a representative characteristic of the profound overlap between brain pathways that: (1) modulate wakefulness; (2) interpret emotional information; and (3) calibrate motivated behaviors. Interestingly, many emotional arousal circuits communicate using specialized signaling molecules called *neuropeptides* to broadly modify neural network activities. One major neuropeptide-enriched brain region that is critical for emotional processing and has been recently implicated in sleep regulation is the bed nuclei of stria terminalis (BNST), a core component of the *extended amygdala* (an anatomical term that also includes the central and medial amygdalae, nucleus accumbens shell, and transition zones betwixt). The BNST encompasses an astonishing diversity of cell types that differ across many features including spatial organization, molecular signature, biological sex and hormonal milieu, synaptic input, axonal output, neurophysiological communication mode, and functional role. Given this tremendous complexity, comprehensive elucidation of the BNST neuropeptide circuit mechanisms underlying emotional arousal presents an ambitious set of challenges. In this review, we describe how rigorous investigation of these unresolved questions may reveal key insights to enhancing psychiatric treatments and global psychological wellbeing.

## Introduction

Precise control of wakefulness (“arousal”) is essential for generating the adaptive forms of reward-seeking and stress resilience that encourage healthy survival (Tsujino and Sakurai, 2009; Eban-Rothschild et al., 2017). Thus, neuronal wakefulness systems were shaped by evolution to confer high sensitivity for detecting, interpreting, and acting upon emotional stimuli. Healthy sleep/wake cycles are essential for optimal cognition and emotion, and poor sleep quality can lead to deleterious changes in the physiological function of brain circuits that gate behavioral responses to emotional stimuli (Koob and Colrain, 2020). In mental health conditions of addiction, anxiety, and depression, maladaptive responses to hedonically-valenced stimuli are linked to distinct activity patterns in brain arousal pathways. Indeed, stress is a major factor driving *insomnia* (inability to sleep), and various sleep-related disturbances are common among individuals enduring stress-related psychiatric conditions. On the other hand, experiencing pleasure and anticipating future reward can also extend wakefulness and prevent healthy sleep, highlighting the ability of both positive and negative hedonically-valenced stimuli to shift the thresholds of arousal (Eban-Rothschild et al., 2018). A further fascinating example of the link between emotion and arousal is the sleep disorder *narcolepsy with cataplexy*, in which powerful feelings of euphoria or aversion can interrupt wakefulness by triggering rapid intrusion of a sleep-like state (Adamantidis et al., 2020). These profound neuroscientific mysteries hint at the commonalities among (and/or interactions between) brain pathways that calibrate wakefulness, process emotional information, and generate motivated behaviors.

Intriguingly, many emotional arousal circuits use specialized modulatory signaling molecules called *neuropeptides* to fine-tune the coordination of broad neural network activity (Ryabinin et al., 2012; Schank et al., 2012; Giardino and de Lecea, 2014; Kash et al., 2015; Li et al., 2017). One major neuropeptide-enriched emotional processing network is the *extended amygdala* (an anatomical term referring to neurons spanning the bed nuclei of stria terminalis (BNST), central and medial amygdalae (CeA, MeA), nucleus accumbens shell (NAcSh), and the transition zones betwixt; Alheid, 2003). While communication *via* neuropeptide signaling likely allows the BNST to perform sophisticated control of emotional arousal circuitry, the primary mechanisms underlying changes in synthesis, storage, and release of peptide neuromodulators from BNST neurons remain largely undescribed. This is due in part to the complex patterns of more than 10 discrete neuropeptides that are distributed in varying combinations of multi-neuropeptide co-expression amongst up to forty unique cellular subpopulations (Moffitt et al., 2018; Welch et al., 2019; Rodriguez-Romaguera et al., 2020). The BNST encompasses a particularly astonishing diversity of cell types that differ along spectrums of several features, including spatial organization, molecular signature, biological sex and hormonal milieu, synaptic input, axonal output, neurophysiological messaging, and functional role (Kash et al., 2015; Lebow and Chen, 2016; Vranjkovic et al., 2017; Ch’ng et al., 2018; Beyeler and Dabrowska, 2020).

Historically, an all-encompassing framework for the BNST cell groups and connections driving emotional behaviors was limited by existing pharmacological and neurochemical approaches. Recent advances in genetic, optical, and computational tools for mapping, manipulating, and monitoring brain activity have revolutionized functional annotation of behavioral neurocircuits (Saunders et al., 2015; Nectow and Nestler, 2020; Xia and Kheirbek, 2020). Nevertheless, comprehensive elucidation of the BNST neuropeptide mechanisms underlying emotional arousal presents an ambitious set of challenges. In this review, we describe how rigorous investigation of these unresolved questions may reveal key insights to enhancing psychiatric treatments and global psychological wellbeing.

## Spatially-Defined BNST Cell Types

The BNST is a ventromedial forebrain complex surrounded on all sides by the hypothalamus, thalamus, striatum, septum, and lateral ventricles. Given the wide-ranging descriptions of “BNST”, we primarily discuss the multiple distinct neuronal populations corresponding to those encompassed within adult mouse (*Mus musculus*) brain stereotaxic coordinates approximately +0.45 to −0.35 mm anterior/posterior (A/P), 0.40 to 1.20 mm medial/lateral (M/L) bilaterally off the midline, and −4.0 to −5.0 mm dorsal/ventral (D/V). Various systems of nomenclature have been proposed for labeling unique BNST subcompartments, but the classification of BNST cellular populations based solely on spatial location remains unstandardized and highly subjective (Bota et al., 2012; Lebow and Chen, 2016; Barbier et al., 2021). This persisting lack of consensus for definitive BNST spatial subdivisions reflects the challenges faced by early anatomists, who first divided BNST on the M/L axis, only to be challenged by developmental biologists who inferred a predominantly A/P axis, followed by synaptic physiologists, neurochemists, and others who emphasized a D/V axis (corresponding to divergent patterns of monoaminergic innervation, for example; De Olmos and Ingram, 1972; Krettek and Price, 1978; Weller and Smith, 1982; Bayer and Altman, 1987; Dong et al., 2000; Egli and Winder, 2003; Bota et al., 2012; McElligott et al., 2013; Radley and Johnson, 2018). Although functional associations with BNST divisions across each of the anatomical axes have sparked valuable hypotheses, variability in the degree to which unique BNST features differ across distinct spatial dimensions limits the holistic impact of relying solely on such descriptors to functionally parcellate the BNST.

For example, the term “ventral BNST” commonly refers to neurons located directly ventral to (beneath) the anterior commissure (a prominent white matter tract that forms a wide horizontal band when viewed in the coronal plane). However, pioneering neuroanatomists acknowledged more than 30 years ago that, while the commissure may be a useful landmark for dividing general areas of the BNST, “it does not necessarily always define strict cytoarchitectonic boundaries, since a component of the dorsal area may well be separated and come to lie in the ventral area” (Ju and Swanson, 1989). In other words, the commissure forms a wide horizontal shape only at certain points along the rodent BNST A/P axis, and the commissure’s departure from view in the caudal BNST reveals contiguous cellular populations that may have been “divided” on a D/V axis in rostral sections purely incidentally. Indeed, Ju and Swanson (1989) noted that “Immunohistochemical studies with antisera to several peptides also indicate very similar staining patterns within these (D/V) regions. It seems clear to us, therefore, that the anterior commissure *simply passes through* the BNST” (Ju and Swanson, 1989).

Upon eschewing the commissure as a monolithic landmark, Swanson and colleagues identified at least five different BNST cellular populations residing ventrally to the commissure (Ju and Swanson, 1989; Ju et al., 1989; Dong et al., 2001a,b; Dong and Swanson, 2003, 2004a,b, 2006a,b,c). Although they originally used the abbreviation “vBNST” to refer only to a particular ventralmost subnucleus within the ventral BNST complex (Ju and Swanson, 1989; Ju et al., 1989; Dong et al., 2000, 2001a,b; Dong and Swanson, 2003, 2004a,b, 2006a,b,c), modern widespread usage of the term “vBNST” generally translates to “ventral BNST writ large”, and the commissure remains a major dividing line for ascribing any readily identifiable characteristics that may be distinguished along the D/V BNST axis. Supplementary Table 1 displays the incongruity of stereotaxic coordinates used to target the mouse “ventral BNST” in recent behavioral neuroscience publications, reflecting the limitations of relying on ambiguous spatial descriptors (Jennings et al., 2013b; Dedic et al., 2018; Kim et al., 2018; Hardaway et al., 2019; Chen et al., 2020; Girven et al., 2020). Especially given the renewed interest in adjacent bordering structures (i.e., ventral pallidum, substantia innominata, preoptic area; McHenry et al., 2017; Gordon-Fennell et al., 2019; Ottenheimer et al., 2019; Stephenson-Jones et al., 2020), investigators may decide to refine their definitions when examining ventrally-located BNST neuronal populations. Of course, “ventral BNST” is simply one of many instances of imperfect BNST anatomical nomenclature. Numerous additional examples of incongruous systems for spatially labeling neuronal subtypes serve only to further strengthen the rationale for adopting a BNST framework that heavily incorporates non-spatial defining features (Ju and Swanson, 1989; Jennings et al., 2013a, b; Kim et al., 2013; Giardino et al., 2018; Barbier et al., 2021).

Although beyond the scope of this review, potential differences in the spatial organization of BNST cell types between various rodent and primate species also require serious consideration. In addition to the anatomical literature cited above, we refer the reader to foundational work from Bales, Blackford, Fox, Fudge, Luyten, Shackman, Trainor, Zahm, and others (Zahm, 1998; Fudge and Haber, 2001; Zahm et al., 2003; Hostetler et al., 2011; Avery et al., 2014, 2016; Fox et al., 2015; Luyten et al., 2016; Shackman and Fox, 2016; Fudge et al., 2017; Oler et al., 2017; Raymaekers et al., 2017; Reichard et al., 2017; Theiss et al., 2017; Duque-Wilckens et al., 2018; Fox and Shackman, 2019; Luyck et al., 2019, 2020; Flook et al., 2020; Luyten, 2020). Indeed, most *in vivo* data generated from monkey and human BNST thus far has been collected using methods with a low spatial resolution like functional magnetic resonance imaging and deep brain stimulation. Within these mental health contexts, reliable *in vivo* parcellation of human BNST subcompartments remains a lofty goal. Keeping this in mind, we encourage others to acknowledge the possibility that acquiring enhanced spatial resolution of the BNST in human patients may turn out to be largely inconsequential for improving overall psychiatric and neurological outcomes. In doing so, we posit that emphasis on *non-spatial* aspects of BNST neurons (such as molecular markers, physiological features, long-range projection targets, and sources of upstream neural inputs) may hold the key for accelerating discovery on functional contributions of BNST circuitry to behavior and sleep/wake arousal states.

## Molecularly-Defined BNST Cell Types

**Figure 1:**
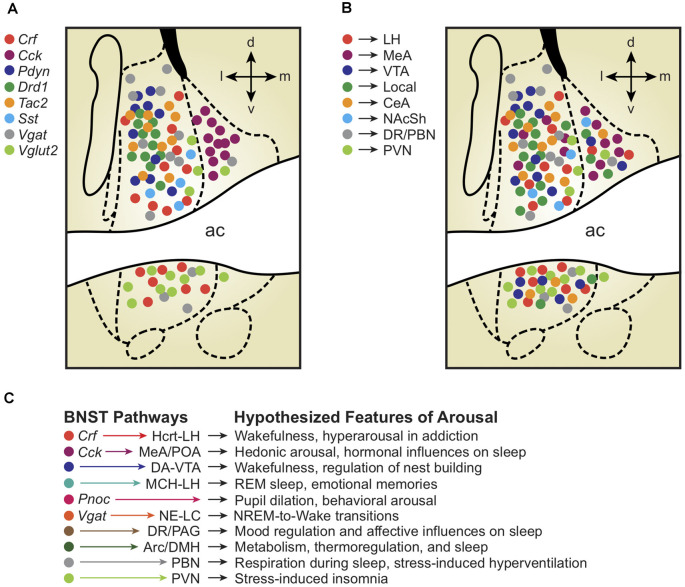
**(A)** Depiction of molecularly-defined bed nuclei of stria terminalis (BNST) cell types and their distribution across BNST subregions. Note the remarkable compartmentalization of some cell-types compared with others (*Cck* vs. *Crf* vs. *Vglut2*). **(B)** Depiction of projection-defined BNST cell types and their approximate distributions across BNST subregions. Interestingly, some (but not all) projection-defined cell types roughly map onto corresponding molecularly-defined subpopulations. **(C)** Depiction of BNST cell types, pathways, and their hypothesized relationships with distinct features of arousal and sleep/wake regulation. Colors of each hypothesized pathway reflect the molecularly- and projection-defined BNST cell types when consistent with panels **(A)** and **(B)**. Distinct colors represent hypothesized BNST pathways with unknown molecular traits and spatial distributions across the BNST.

Similar to the rest of the extended amygdala, the BNST contains many cell types marked by a myriad of neurotransmitters, neuropeptides, receptors, enzymes, and regulatory proteins (Bota et al., 2012). The BNST primarily consists of subpopulations of inhibitory neurons marked by the GABA transporter *Vgat*, as well as excitatory populations marked by the glutamate transporter *Vglut2*, and a mixed excitatory/inhibitory population marked by co-expression of *Vgat* and *Vglut3* (Kudo et al., 2012; Jennings et al., 2013b). Although these various GABAergic and glutamatergic populations are widely dispersed, mapping molecularly-defined cell types to different spatial areas of the BNST may help clarify how different subregions regulate emotional arousal behaviors (Figure 1).

To survey BNST neurons defined by molecular markers other than GABA/glutamate transporters, multiple studies first used Cre driver rodent lines with Ai9/Ai14 tdTomato fluorescent reporter mice or viral labeling techniques and combined anatomical analyses with immunohistochemical staining (Chen et al., 2015; Pomrenze et al., 2015; Nguyen et al., 2016; Giardino et al., 2018; Walker et al., 2019). The global population of *Vgat*-BNST neurons was found to encompass several molecularly-defined subgroups, including neurons expressing genetic and protein markers for the neuropeptides corticotropin-releasing factor (*Crf*; Dabrowska et al., 2013, 2016) and cholecystokinin (*Cck*; Giardino et al., 2018). *Crf* and *Cck* are non-overlapping GABAergic subpopulations that occupy separate lateral vs. medial subdivisions, co-residing in adjacent compartments throughout the middle of the BNST A/P axis, approximately +0.2 to −0.2 mm from Bregma in the mouse. In addition to *Crf* and *Cck*, the neuropeptides dynorphin (*Pdyn*), enkephalin (*Penk*), neurotensin (*Nts*), neuropeptide Y (*Npy*), nociceptin (*Pnoc*), somatostatin (*Sst*), substance P (*Tac1*), neurokinin B (*Tac2*), and vasopressin (*Avp*) are also found in neurons throughout the BNST (Malsbury and McKay, 1987; Walter et al., 1991; Poulin et al., 2009; Kudo et al., 2014; Crowley et al., 2016; Ahrens et al., 2018; Giardino et al., 2018; Zelikowsky et al., 2018; Kovner et al., 2019; Rigney et al., 2019; Rodriguez-Romaguera et al., 2020; Smith et al., 2020; Whylings et al., 2020; Xiao et al., 2020; Figure 1A). Labeling for the calcium-binding protein calretinin appears selective for the dorsolateral (dl)BNST, whereas dopamine receptor type-1 (*Drd1*) and protein kinase C delta (*Pkcd*) neurons cluster more specifically within the oval nucleus (ovBNST, a discrete subnucleus within the larger dlBNST subregion; Kim et al., 2013; Nguyen et al., 2016; Pomrenze et al., 2019a; Wang et al., 2019).

Breakthrough efforts to characterize the entire genetic diversity of the BNST and surrounding regions at the single-cell level provided evidence for up to 37 distinct neuronal subtypes, although an exhaustive discussion of this data is beyond the scope of our review (Moffitt et al., 2018; Welch et al., 2019). A more recent single-cell RNA sequencing study targeted specifically in the dorsal (d)BNST identified several neuronal clusters, including those marked by expected genes (e.g., *Pkcd*, *Sst*, *Npy*), but also some surprising markers (e.g., *Lmo4*; Rodriguez-Romaguera et al., 2020).

Numerous BNST neurons (particularly in the posteromedial [pm]BNST) express markers for actions of gonadal steroid hormones, including the androgen receptor (AR), progesterone receptor (PR), estrogen receptors, and aromatase (Aro), the enzyme that converts androgens to estrogens (Bayless and Shah, 2016). Aro-BNST neurons have been well-studied in contexts of sexually dimorphic behaviors (Bayless et al., 2019), and pmBNST neurons expressing AR and PR are more numerous in males vs. females (Juntti et al., 2010; Yang et al., 2013), highlighting the importance of sex differences and hormonal interactions when studying BNST contributions to emotional arousal (Bangasser and Shors, 2008; Bangasser et al., 2019).

Concerning the potential wake-modulating effects of molecularly-defined BNST subpopulations, the reported pupil dilatory effects of *Pnoc*-BNST neuron stimulation (Rodriguez-Romaguera et al., 2020) suggest that such rapid arousal responses may also regulate sleep/wake state transitions, but this has not been explicitly tested. Furthermore, while genetic markers in the BNST have thus far been *primarily used to simply define the type of cell* rather than determine the role of the corresponding protein product, future progress will shift toward understanding the physiological actions of the molecule itself. For example, it will be essential to assess whether modern neurotechnological approaches used to *stimulate CRF-expressing BNST neurons* will be sufficient to accurately recapitulate the sleep/wake changes resulting from the *natural release of the CRF neuropeptide* *from BNST neurons*.

## Projection-Defined BNST Cell Types

A major property of the BNST is its connections with a plethora of downstream target brain regions, providing a useful framework for conceptualizing the potential contributions of BNST neurons to distinct features of sleep/wake arousal states (Figure 1B). For example, by examining BNST projection neurons that form synapses with cells in limbic regions regulating emotional behavior, One landmark article characterized the role of three different BNST pathways in separate features of anxiety (Kim et al., 2013). The authors showed that neurons in the anterodorsal (ad)BNST target the lateral hypothalamus (LH), ventral tegmental area (VTA), and parabrachial nucleus (PBN) to drive anxiolysis, reward, and decreased respiratory rate, respectively. Retrograde tracing determined minimal overlap between cells projecting to the three different downstream regions, collectively implying unique anxiety-relevant functions for different BNST cell types based in part on their projection targets. Given the rich literature describing roles for the LH (Li et al., 2017, 2018), VTA (Eban-Rothschild et al., 2016, 2020), and PBN (Kaur and Saper, 2019) in various aspects of sleep/wake regulation, we speculate that such BNST axonal outputs controlling separable components of emotional behavior may also modulate distinct physiological aspects of sleep/wake arousal states (Figure 1C).

In earlier studies of BNST neuroanatomy in rodents, VTA-projecting BNST neurons had been characterized by the Watanabe group, who identified a double-inhibitory pathway in which GABAergic BNST neurons preferentially target VTA-GABA neurons (Kudo et al., 2012). Kudo et al. (2012, 2014) also identified VTA-projecting BNST neurons marked by the glutamate transporters *Vglut2* and *Vglut3*, as well as the opioid peptide *Penk*, providing multiple diverse mechanisms for modulating the activity of GABA and dopamine (DA) neurons in the VTA. Jennings et al. (2013b) reported that *Vgat* and *Vglut2* neurons specifically in the ventral (v)BNST synapse onto GABA and DA VTA neurons, where they control distinct motivational states. Numerous other studies also focused on VTA-projecting BNST neurons, including those labeled by *Crf* and *Pdyn* (Briand et al., 2010; Silberman et al., 2013; Marcinkiewcz et al., 2016; Pina and Cunningham, 2017; Rinker et al., 2017; Companion and Thiele, 2018; Dedic et al., 2018; Fellinger et al., 2020).

Given recent evidence that VTA-DA neurons drive wakefulness and regulate nest-building (a critical sleep preparatory sequence in mice), BNST→VTA-DA circuits may therefore be a key mechanism influencing ethologically-relevant behavioral arousal (Eban-Rothschild et al., 2016, 2017; Eban-Rothschild and de Lecea, 2017). Furthermore, the activity of the VTA-GABA neuron population was found to be positively correlated with high-frequency gamma signal (30–80 Hz) during wakefulness, providing another measure of physiological arousal likely impacted by BNST→VTA projections (Eban-Rothschild et al., 2020).

Separate efforts on LH-projecting neurons identified a large *Vgat-*BNST population that preferentially targeted *Vglut2*-LH neurons downstream (Jennings et al., 2013a). Later studies determined that LH-projecting *Vgat-*BNST neurons include both *Crf* and *Cck* subpopulations that exhibit divergent preferences for downstream target cell types, with *Crf*-BNST neurons displaying a particularly high level of connectivity with LH neurons containing the arousal-promoting neuropeptide hypocretin (*Hcrt*; also known as orexin; Giardino et al., 2018). *Hcrt*-LH neurons comprise a subset of glutamatergic LH neurons, consistent with the interpretation that *Crf*-BNST→*Hcrt*-LH connections represent a subset of the larger *Vgat*-BNST→*Vglut2*-LH pathway. González et al. (2016) also investigated BNST→LH connectivity, finding that *Vgat*-BNST neurons synapse onto both Hcrt/orexin and melanin-concentrating hormone (MCH) neurons of the LH. This discovery of BNST neurons interacting with the MCH system is notable based on several studies showing that *Mch*-LH neurons promote rapid eye movement (REM) sleep (Bandaru et al., 2020).

In addition to their LH projections, *Crf* and *Cck* BNST neurons innervate (to varying degrees) the paraventricular nucleus of the hypothalamus (PVN), medial amygdala (MeA), CeA, medial preoptic area (mPOA), NAcSh, ventral premammillary nucleus (PMv), and ventrolateral periaqueductal gray (vlPAG; Dabrowska et al., 2016; Giardino et al., 2018). BNST projections to the PVN are particularly relevant given recent findings that glutamatergic neurons in this region are critical for the control of wakefulness (Liu et al., 2020). Further pursuing questions of BNST→hypothalamus connectivity, Barbier et al. (2021) traced the long-range projections of dlBNST and dorsomedial (dm)BNST neurons in exquisite detail, finding that the dlBNST projects especially to the LH and tuberomammillary nucleus (TMN; the site of wake-promoting histamine neurons; Barbier et al., 2021). In contrast to the dlBNST, the dmBNST preferentially innervates the PVN, the arcuate nucleus (Arc), and dorsomedial hypothalamus (DMH) regions that may influence sleep pressure *via* regulation of metabolic and thermoregulatory processes (Barbier et al., 2021).

In addition to targeted investigations, serendipitous retrograde labeling of upstream neurons has yielded surprising BNST outputs and circuit motifs. For example, monosynaptic rabies tracing from either “patch” or “matrix” neurons in the dorsal striatum revealed a major input from the dBNST to patch neurons that were largely absent from matrix neurons (Smith et al., 2016). These dBNST neurons form inhibitory synapses with striatal patch neurons who in turn project to DA neurons in the substantia nigra. Similarly, a GABAergic projection from *Sst*-BNST neurons was recently characterized and shown to form synapses with parvalbumin interneurons in the NAcSh (Xiao et al., 2020). Therefore, in addition to directly targeting the VTA, the BNST is capable of modulating mesolimbic DA function through indirect mechanisms at sites of midbrain innervation in the striatum.

Monosynaptic rabies tracing also identified BNST neurons that project directly to serotonin and GABA neurons in the dorsal raphé nucleus (DR; Weissbourd et al., 2014). Interestingly, BNST→DRN neurons preferentially target GABA cells over serotonin cells. While the majority of the BNST→DR cells were GABAergic at mouse coordinates +0.14 mm A/P from Bregma, a smaller set of BNST→DR neurons were labeled by *Vglut2* at +0.02 mm A/P from Bregma. Separate rabies tracing studies also revealed BNST inputs to noradrenergic locus coeruleus neurons (Schwarz et al., 2015), DA and GABA VTA neurons (Beier et al., 2015), and CRF receptor 1 (CRFR1) PVN neurons (Jiang et al., 2018).

Beyond its rich collection of long-range projection neurons, the BNST contains several species of short-range interneurons. The Deisseroth group showed that ovBNST neurons project locally between subregions of the adBNST where they release GABA (Kim et al., 2013). In line with this, ovBNST neurons secrete dynorphin to inhibit excitatory basolateral amygdala (BLA) fibers innervating the adBNST (Crowley et al., 2016). Neurons in ovBNST also send dense axons to the vBNST (Dong et al., 2001; Wang et al., 2019). Aside from inhibitory local connections, many studies raised the possibility that neuropeptide modulators are also released locally from within the BNST. For example, NPY enhances inhibitory transmission in *Crf*-BNST neurons (Pleil et al., 2015) and although this study did not identify the source of NPY, the BNST contains several NPY-expressing neurons capable of local release. Another study from the Kash group found a complex microcircuit in the BNST comprised of *Crf* interneurons that are modulated by serotonin and regulate the activity of separate long-range BNST projection neurons (Marcinkiewcz et al., 2016). Collectively, these studies demonstrate the complexity of both long-range and local connectivity in the BNST and point to another dimension of inherent organization arising from gene expression and neurotransmitter phenotype.

## Physiology-Defined BNST Cell Types

The BNST contains multiple neuronal cell types that have been classified according to their electrophysiological properties, most prominently in rats by Hammack and others (Hammack et al., 2007; Hazra et al., 2011; Dabrowska et al., 2013; Rodríguez-Sierra et al., 2013; Silberman et al., 2013; Nagano et al., 2015; Yamauchi et al., 2018). While originally described primarily within the ovBNST, these three types (Type I, II, and III) have also been identified in the non-oval areas of adBNST, as well as anteroventral (av)BNST. Type I neurons exhibit an I_h_-like current in response to hyperpolarizing current injection and a regular firing pattern in response to depolarizing current. Type II neurons exhibit a similar I_h_-like current but burst fire in response to depolarizing current. Type III neurons do not exhibit an I_h_-like current but instead, show a fast rectification in response to hyperpolarizing current and exhibit a regular firing pattern when depolarized. Coupled with the diverse synaptic inputs, these physiological characteristics likely play a strong role in modulating varied forms of BNST output.

Regarding distinct output modes of neurophysiological communication, most outgoing physiological signals from the BNST have been recorded simply as GABAergic/glutamatergic inhibitory/excitatory postsynaptic currents. However, the existence of co-expressed neuropeptides in *Vgat*-BNST neurons suggests the likelihood of multiplexed modes of signaling in which single cells may influence downstream activity *via* a combination of slow-acting neuropeptide release and fast-acting classical neurotransmitter release. Indeed, a growing suite of fluorescent sensor tools for detecting receptor signaling with the cell-type resolution at rapid timescales may prove revolutionary for elucidating the sleep/wake mechanisms of neuropeptide release and actions across synaptic and extrasynaptic sites of the BNST circuitry (Gizowski et al., 2016; Patriarchi et al., 2018; Sun et al., 2018).

## Functionally-Defined BNST Cell Types

### Hedonic Valence

The BNST is known to impact both positive and negative states of reward and stress, and early studies relied primarily on electrolytic and neurochemical lesions to generate several opposing hypotheses regarding the bi-valent emotional behaviors generated by activity in the BNST (Bangasser et al., 2005; Pezuk et al., 2008; Resstel et al., 2008). Access to molecularly-defined cell types enabled by modern neurotechnology has ushered in several recent advances in understanding the sources of hedonic valence in the BNST, beginning with studies from the Stuber group showing that optical stimulation of *Vgat*-BNST and *Vglut2*-BNST neurons produced approach and avoidance behaviors, respectively (Jennings et al., 2013a, b).

Going beyond the separation of large populations of GABAergic vs. glutamatergic neurons, additional tools for recording and modulating cell-specific neural activity *in vivo* revealed that dlBNST *Crf* neurons preferentially respond to aversive stimuli and drive behavioral avoidance, whereas dmBNST *Cck* neurons are activated by rewarding stimuli and generate behavioral preference/approach (Giardino et al., 2018). Mirroring this lateral/medial functional distinction, *Drd1* neurons in the ovBNST/dlBNST and *Six3* neurons in the dmBNST also drive avoidance and approach, respectively (Giardino et al., 2018). Because activation of the global *Vgat*-BNST population promotes positive valence (Jennings et al., 2013a, b; Giardino et al., 2018), we hypothesize that *Crf* marks a specialized subgroup of lateral *Vgat*-BNST neurons with opposing functional properties from the larger set of combined lateral and medial *Vgat*-BNST cells. In other words, global *Vgat*-BNST stimulation may more closely resemble activation of medial *Vgat-*BNST neurons (like *Cck*) rather than lateral *Vgat-*BNST neurons (like *Crf*).

It should be noted that the vast majority of data on BNST circuits driving hedonic valence has been generated only from male mice. Given that the BNST is a major site for integrating signals from centrally-circulating gonadal steroids, intense investigation of hormonal influences on the function of extended amygdala pathways may be key for understanding sex differences in the stress response and reward sensitivity. Future studies may also seek to titrate levels of stressor exposure, drug consumption, and other variables to determine whether such experiential factors can influence the hedonic valence associated with mobilization of particular BNST subpopulations.

### Anxiety

Despite the distinct hedonic valences associated with stimulating *Crf* and *Cck* BNST neurons, optogenetic or chemogenetic activation of either *Crf* or *Cck* BNST neurons led to increased indices of anxiety-like behavior in multiple paradigms (Giardino et al., 2018), suggesting that standard measures of “anxiety” in rodents may reflect generalized arousal states independent of hedonic valence *per se*. Consistent with this interpretation, activation of *Vgat*-BNST neurons (Mazzone et al., 2018), *Drd1*-ovBNST neurons (Kim et al., 2013), *Pdyn*-BNST→VTA neurons (Fellinger et al., 2020), dlBNST→CeA neurons (Yamauchi et al., 2018), and *Crf*-CeA→dlBNST neurons (Pomrenze et al., 2019b) all increased anxiety-like behaviors. Yet, separate studies found that stimulation of adBNST→LH neurons (Kim et al., 2013), *Vgat*-vBNST→VTA neurons (Jennings et al., 2013b), and *Sst*-BNST→NAcSh neurons (Xiao et al., 2020) had the opposite effect of reducing anxiety, revealing some unresolved issues regarding how BNST microcircuits and long-range pathways regulate stress-related “anxiety” phenotypes. Anxiogenic circumstances like fear learning and stress-induced social deficits also strongly engage the BNST (Bjorni et al., 2020; Emmons et al., 2021), providing multiple perspectives for approaching the study of BNST in anxiety-related emotional arousal.

## The BNST in Sleep, Wake, and Emotional Arousal

Only a handful of studies have explicitly investigated the BNST within the context of sleep/wake arousal states and corresponding neurophysiological rhythms. Beginning in 1995, *in vivo* recordings of 63 single units in the BNST of cats revealed that 72% of neurons fired more frequently during wakefulness and rapid eye movement (REM) sleep than during “quiet sleep” (presumably non-REM/slow-wave sleep; NREM; Terreberry et al., 1995). These findings are consistent with more recent data indicating that excitatory projections from the BNST may activate REM-active neurons in the sublaterodorsal tegmental nucleus (SLD) of the brainstem (Boissard et al., 2003; Rodrigo-Angulo et al., 2008). In the BNST of rats, sleep deprivation increased cFos and CRF protein expression (Duan et al., 2005; Deurveilher et al., 2008) and M3 muscarinic acetylcholine receptor gene expression (Kushida et al., 1995), suggesting possible neuropeptidergic and cholinergic mechanisms for homeostatic sleep drive in the BNST.

In 2014, an intriguing arousal-related physiological signature was discovered in the extended amygdala by Haufler and Pare (2014), who recorded high-frequency oscillations (HFOs; 110–160 Hz local field potentials) that appeared with a significantly higher incidence in the BNST and CeA relative to surrounding areas (striatum, pallidum, septum; Haufler and Pare, 2014). HFOs in hippocampal, cortical, and subthalamic brain regions are thought to have functional consequences in memory-processing, epileptic seizures, and symptoms of Parkinson’s disease, respectively. Whereas the role of HFOs in the BNST remains unclear, their ability to entrain large populations of neurons (with greater power during REM vs. non-REM sleep; Haufler and Pare, 2014) provides a fascinating example of the network-level changes in extended amygdala activity that might profoundly influence discrete states of arousal. Future endeavors will revolutionize understanding of how arousal state transitions may be aligned to physiological activity changes and/or receptor signaling events in a BNST cell type-specific manner by implementing combinatorial approaches like EEG/EMG sleep monitoring in tandem with next-generation neural recording technologies (fiber photometry, miniscope, and two-photon imaging).

To directly investigate the BNST as a potential node in the arousal circuitry, Kodani et al. (2017, 2019) utilized optogenetic methods and reported that they could generate immediate transitions from NREM sleep to wakefulness by stimulating the global GABAergic BNST population in *Gad67*-Cre mice. *Gad67*-BNST arousal was associated with activation of norepinephrine neurons in the locus coeruleus (NE-LC), an established wakefulness-promoting center. *Gad67*-BNST arousal was also associated with activation of Hcrt-LH neurons and required signaling of Hcrt receptors (Kodani et al., 2017), consistent with the reported contributions of BNST→LH circuits to emotional arousal and related behaviors (Giardino and de Lecea, 2014; González et al., 2016; Giardino et al., 2018; Barbier et al., 2021). Of course, the precise directionalities of the relationships between endogenous BNST activity, emotional behaviors, and changes in sleep and wakefulness remain mostly uncharacterized. For example, whereas states of stress might be hypothesized to drive insomnia *via* chronically elevated BNST hyperactivity, additional experiments are required to determine whether excess BNST activity is truly a contributing factor or rather an indirect consequence of stress-induced arousal.

In addition to providing *outputs* to wake-promoting downstream targets in the LH, VTA, and PVN, the BNST also receives *inputs* from arousal-promoting neurons such as calretinin cells of the paraventricular thalamus (PVT; Hua et al., 2018). Hua et al. (2018) recently found that starvation promotes a state of hyperarousal *via* activation of the PVT-calretinin circuit, which can be blocked by chemogenetic inhibition of the PVT→BNST pathway. Despite this exciting progress, a complete understanding of the arousal-promoting abilities of the global GABAergic BNST population requires additional studies. Future experiments will aim to determine whether distinct molecularly-defined or pathway-specific BNST outputs are necessary and/or sufficient for regulating various forms of behavioral arousal and natural sleep-to-wake transitions (see hypothesized functions in Figure 1C).

On this note, it will be important to causally determine which BNST neurocircuits have experience-dependent effects on sleep/wake as a function of negative vs. positive hedonic valence. This can be accomplished by monitoring the activities of extended amygdala pathways and corresponding changes in arousal following emotional experiences with ethologically aversive or rewarding stimuli. For example, the discovery that reward-promoting Cck-BNST neurons are densely connected with the medial amygdala (MeA; Giardino et al., 2018) suggests that these pathways powerfully influence arousal changes linked to innately rewarding consummatory behaviors. In contrast to the Cck-BNST→MeA circuit, Crf-BNST→Hcrt-LH connections likely drive stress-induced insomnia following psychosocial challenges.

Given the well-established role of the BNST in drug-seeking behavior (Vranjkovic et al., 2017), attention should be placed on the idea that addiction-related sleep disturbances are driven by specific extended amygdala pathways. Core features of drug addiction (e.g., craving, relapse, and withdrawal) are each associated with maladaptive neuroplasticity in homeostatic circuits that regulate sleep/wake cycles and stress sensitivity, illustrating how addiction can be viewed as a condition of pathological hyperarousal (Koob, 2013; Koob and Colrain, 2020). Substance use disorders are highly co-morbid with other arousal-related psychiatric conditions (e.g., insomnia, anxiety, PTSD, panic), and the BNST may be a common substrate linking dysregulated hedonic processing to chronic sleep disruption.

Finally, the BNST may be an important and overlooked link in emotional arousal about the sleep disorder *narcolepsy with cataplexy* (in which powerful feelings of euphoria or aversion can interrupt wakefulness by triggering rapid intrusion of a sleep-like state). Intriguingly, narcolepsy is associated with selective loss of Hcrt neurons in the LH. Based on existing evidence for BNST→LH connections driving motivated behaviors, detailed studies are warranted on BNST circuit contributions to emotionally-triggered arousal destabilization. Of course, the existence of putative sleep-promoting BNST neurons remains to be seen. However, BNST connections with hypothalamic nuclei regulating the release of prolactin, oxytocin, and vasopressin suggest that experiments linking BNST activity to arousal changes following binge eating (“food coma”) and sexual behavior (mating) may be successful in revealing the neurocircuitry of mysterious phenomena like post-ingestive sleep, post-copulatory sleep, and narcolepsy with cataplexy.

## Conclusions

Altogether, our understanding of extended amygdala circuits regulating affective behaviors will have direct relevance to therapeutic strategies aimed at modulating motivation and sleep/wake regulation. Before the advent of tools permitting cell type-specific and pathway-specific labeling and manipulation, the inherent complexity of extended amygdala pathways hindered progress in understanding their roles in emotional arousal. As BNST circuits continue to be disentangled at the genetic, synaptic, and systems levels, new anatomical and functional frameworks are taking shape that illustrates the multifaceted nature of the BNST. Data of this kind are extremely valuable for developing new therapeutic approaches for a range of neuropsychiatric conditions, highlighting the extraordinary relevance of emotional arousal circuits to researchers and clinicians across the fields of neuroscience, genetics, psychology, and mental health interventions. We anticipate that as studies on BNST function become more precise, effective clinical treatment strategies will be successfully developed.

## Author Contributions

All authors listed have made a substantial, direct and intellectual contribution to the work, and approved it for publication.

## Conflict of Interest

The authors declare that the research was conducted in the absence of any commercial or financial relationships that could be construed as a potential conflict of interest.
