# Supplementary material for: Extended Amygdala Neuropeptide Circuitry of Emotional Arousal: Waking Up on the Wrong Side of the Bed Nuclei of Stria Terminalis
Source: Front Behav Neurosci. 2021 Feb 9;15:613025. doi: 10.3389/fnbeh.2021.613025 (PMC7900561; doi:10.3389/fnbeh.2021.613025)
Supplement: Supplementary file 1 [file Table_1.DOCX]

**Table 1. Stereotaxic coordinates for targeting “ventral BNST” in male mice on a C57BL/6J background.**

Taken from representative behavioral neuroscience studies in adult mice published between 2013 and 2020. Although D/V coordinates are consistent, targeting varies >1.2mm along the A/P axis and >0.5mm along the M/L axis.

| **Citation** | **A/P** | **M/L** | **D/V** |
| --- | --- | --- | --- |
| Kim et al., 2018^1^ | +1.00 mm | +/- 1.00 mm | -4.70 mm |
| Hardaway et al., 2018^2^ | +0.20 mm | +/- 1.05 mm | -4.70 mm |
| Girven et al., 2020^3^ | +0.16 mm | +/- 0.90 mm | -4.80 mm |
| Dedic et al., 2018^4^ | +0.15 mm | +/- 0.80 mm | -4.75 mm |
| Jennings et al., 2013^5^ | +0.14 mm | +/- 0.90 mm | -4.80 mm |
| Chen et al., 2020^6^ | -0.24 mm | +/- 0.50 mm | -4.70 mm |

**Table References**

1. Kim, B. *et al.* Dopamine D2 receptor-mediated circuit from the central amygdala to the bed nucleus of the stria terminalis regulates impulsive behavior. *Proc Natl Acad Sci U S A* **115**, E10730-E10739 (2018).

2. Hardaway, J.A. *et al.* Central Amygdala Prepronociceptin-Expressing Neurons Mediate Palatable Food Consumption and Reward. *Neuron* **102**, 1088 (2019).

3. Girven, K.S. *et al.* Glutamatergic input from the insula to the ventral bed nucleus of the stria terminalis controls reward-related behavior. *Addict Biol*, e12961 (2020).

4. Dedic, N. *et al.* Chronic CRH depletion from GABAergic, long-range projection neurons in the extended amygdala reduces dopamine release and increases anxiety. *Nat Neurosci* **21**, 803-807 (2018).

5. Jennings, J.H. *et al.* Distinct extended amygdala circuits for divergent motivational states. *Nature* **496**, 224-8 (2013).

6. Chen, A.X. *et al.* Specific Hypothalamic Neurons Required for Sensing Conspecific Male Cues Relevant to Inter-male Aggression. *Neuron* (2020).
